# A New Endemic Locality of *Dermacentor reticulatus* in Central–Southern Poland and Its Potential Epidemiological Implications

**DOI:** 10.3390/insects15080580

**Published:** 2024-07-30

**Authors:** Marek Asman, Katarzyna Bartosik, Justyna Jakubas-Zawalska, Agata Świętek, Joanna Witecka

**Affiliations:** 1 Department of Medical and Molecular Biology, Faculty of Medical Sciences in Zabrze, Medical University of Silesia in Katowice, Jordana 19 St., 41-808 Zabrze, Poland; agata.swietek@sum.edu.pl; 2 Department of Biology and Parasitology, Chair of Pharmacology and Biology, Faculty of Health Sciences, Medical University of Lublin, Radziwiłłowska 11 St., 20-080 Lublin, Poland; 3 Sanitary-Epidemiological Station in Świnoujście, Dąbrowskiego 4 St., 72-600 Świnoujście, Poland; jakubas.zawalska@gmail.com; 4 Silesia LabMed Research and Implementation Centre, Medical University of Silesia in Katowice, 19 Jordana St., 41-808 Zabrze, Poland; 5 Department of Parasitology, Faculty of Pharmaceutical Sciences in Sosnowiec, Medical University of Silesia in Katowice, Jedności 8 St., 41-218 Sosnowiec, Poland; jwitecka@sum.edu.pl

**Keywords:** *Dermacentor reticulatus*, *Ixodes ricinus*, tick expansion, tick distribution, tick-borne pathogens, *Borrelia burgdorferi* sensu lato, *Babesia* spp., sympatric tick species

## Abstract

**Simple Summary:**

*Dermacentor reticulatus* is an arthropod vector with great medical and veterinary importance. Its wide distribution and biological characteristics determine its important role in the circulation of pathogens in the parasite–host system. Its occurrence range is divided into western and eastern populations, which are separated from each other by the so-called *Dermacentor*-free zone localized in central Poland. This study aimed to estimate the potential epidemiological significance of *D. reticulatus* in the new endemic focus west of the Vistula River (Upper Silesia, central–southern Poland) and its co-occurrence with *Ixodes ricinus*. The molecular studies revealed the presence of *Rickettsia* spp. in 23.8% of the *D. reticulatus* specimens. In turn, 94.1% of the *I. ricinus* adults were infected with *B. burgdorferi* s.l., 11.7% with *Babesia* spp., and 5.8% with *Rickettsia* spp. Polymicrobial infections were noted in 17.6% of the *I. ricinus*. Our finding emphasizes the risk of infestation by both tick species and the risk of tick-borne infections in an area previously thought to be free of *Dermacentor* ticks. It is necessary to enhance medical and veterinary services for the more efficient diagnosis and prevention of tick-borne diseases in this region.

**Abstract:**

*Dermacentor reticulatus* (Acari: Ixodidae) is an important arthropod vector in medical and veterinary contexts. Its geographic range is divided into western and eastern populations separated by a “*Dermacentor*-free zone” in central Poland. Recent faunistic studies showed a new endemic locality of the species in Upper Silesia to the west of the Vistula River (central–southern Poland) and its co-occurrence with *I. ricinus.* The prevalence of five tick-borne pathogens (TBPs), e.g., *B. burgdorferi* s.l., *Bartonella* spp., *Rickettsia* spp., and *Babesia* spp., in the ticks was assessed with polymerase chain reaction (PCR) methods. The molecular studies revealed the presence of *Rickettsia* spp. in 23.8% of the *D. reticulatus* specimens. In turn, 94.1% of the *I. ricinus* adults were infected with *B. burgdorferi* s.l., 11.7 % with *Babesia* spp., and 5.8% with *Rickettsia* spp. Coinfections with two TBPs were noted in 17.6% of the *I. ricinus*. These findings highlight not only the risk of infestation by both tick species in an area previously considered *Dermacentor*-free, but also the high prevalence of TBPs in the study area. Increased focus on medical and veterinary services appears necessary to diagnose and prevent tick-borne diseases in this region.

## 1. Introduction

Currently, 19 species of ticks are permanent elements of the Polish acarofauna [1]. After *Ixodes ricinus* (Linnaeus, 1758), *Dermacentor reticulatus* (Fabricius, 1794) is the tick species with the second-greatest epidemiological importance [1,2,3]. There are numerous reports on the sympatric occurrence of these species in Poland [4,5,6,7,8]. *Dermacentor reticulatus* inhabits mainly wet mixed forests, meadows, and scrub communities. This tick species is most common in meadows near forest borders and wet forests associated with river valleys, lake shores, and ravine systems. It also occurs in deciduous forests, clearings, forest meadows, and in forest steppes [1,2,9]. There are also literature reports on *D. reticulatus* occurrence in areas with a high degree of anthropopressure [4,6,10,11,12]. *Dermacentor reticulatus* is known to be a competent vector of, e.g., tick-borne encephalitis virus (TBEV), Omsk hemorrhagic fever virus, and spotted fever group (SFG) rickettsia, e.g., *Rickettsia raoultii* and *Rickettsia slovaca*, *Babesia canis*, *Babesia caballi*, and *Theileria equi*, [9,13,14].

The occurrence of various tick species in recently invaded areas is increasingly being observed [15,16,17,18]. Progressive climate change is probably one of the main factors responsible for the changes in their geographic distribution range [15,16,19,20]. Additionally, the loss of forest areas, fluctuations in host population numbers, changes in agricultural land use, and human activity are potential determinants as well [2,21,22]. The shift in the distribution range is particularly evident in the case of *D. reticulatus*, which is gradually being recorded in areas that separate the eastern from western populations of this tick in Europe. Recently, many new endemic areas of the occurrence of this species to the west of the Vistula River have been described in Poland [17,23,24]. Therefore, to protect human and animal health in accordance with the One Health concept, the constant tick expansion and the emergence of new tick-borne disease foci should be monitored. The aim of this study was to estimate the potential epidemiological significance of *D. reticulatus* occurring in a new endemic area in Upper Silesia, central–southern Poland, taking into account its co-occurrence with *I. ricinus*.

## 2. Materials and Methods

### 2.1. Tick Collection and Study Site

Ticks were collected from vegetation in mid-April 2023 with the flagging method [1] between 11.00 a.m. and 1.00 p.m. in selected areas of Sławków (50°30′19.8″ N; 19°40′72.6″ E) 329 m a.s.l. (Upper Silesia, central–southern Poland). The flagging method was used for at least 1 h by one person at each study site. Both an open landscape, e.g., a meadow located close to a mixed forest, and an ecotone near the forest border were the collection sites. The ticks were collected in four plots with a total area of 51 ha located near areas protected under the Natura 2000 Łąki w Sławkowie program PLH 240043. The areas comprise a mosaic of meadow habitats, including *Molinia* meadows, fresh meadows, and wetlands dominated by meadow communities of the orders Molinietalia and Arrhenatheretalia characterized by a very rich flora composition. The most valuable natural communities from the Molinion alliance cover an area of approximately 28.6% of the meadow complex. The valuable natural areas in Sławków, e.g., the ecological corridors of the Biała Przemsza valley, the Sławkowska Struga valley, the Bobrek spring area, small natural marsh areas, and wetlands formed during the construction of rope park facilities, are protected under the provisions of the Nature Protection Act [25].

The tick specimens were placed in the sterile 50 mL polypropylene tubes and stored in 70% ethyl alcohol. Next, the species, developmental stage, and sex of the ticks were identified under the Olympus SZ-40 binocular microscope (Tokyo, Japan) according to the guides to tick identification developed by Siuda [26] and Nowak-Chmura [1]. The risk of tick attacks was assessed using a 5-classed scale proposed by Supergan and Karbowiak [27].

### 2.2. Molecular Analyses

Individual tick specimens were rinsed sequentially in 70% ethanol and sterile ultrapure water to prevent DNA contamination and homogenized using sterile garnet sharp particles, 0.3 mm in diameter (Tissue Grinding Tool, Eurix, Gdańsk, Poland). DNA was isolated from single ticks with the ammonia method [28], and its concentration was measured spectrophotometrically at the 260/280 wavelength using the Implen NanoPhotometer PEARL (Munich, Germany). Then, the samples were frozen at −20 °C and stored for further molecular studies. Tick-borne pathogens (TBPs) were detected in DNA isolates obtained from a single tick with the real-time PCR, PCR, and nested PCR methods. To detect *B. burgdorferi* s.l., real-time PCR analysis was performed using the EURx *Borrelia* qPCR Detection Kit (Gdańsk, Poland) according to the manufacturer’s protocol. The nested PCR and PCR methods were used for the detection of *A. phagocytophilum* and *Babesia* spp., respectively. Two pairs of primers specific to the 16S rRNA gene were applied to detect the presence of *A. phagocytophilum* [29]. For the detection of *Babesia* spp. and *Bartonella* spp. in the ticks, a pair of primers specific to the 18S rRNA gene and the *rpoB* gene, respectively, was used [30,31]. *Rickettsia* spp. were detected with the use of a pair of primers specific to the *gltA* gene [32]. Oligonucleotide primers used in detection of TBPs and PCR conditions are included in Table 1. The amplification products were separated electrophoretically in 2% ethidium bromide-stained agarose gels, visualized under ultraviolet light, and photographed in a Vilber Lourmat device (Collegien, France). Next, *Babesia* spp.-positive samples were isolated from the gels and purified using the EURx GeneMATRIX Agarose-OUT DNA Purification Kit (Gdańsk, Poland) according to the manufacturer’s protocol. Sequencing was performed by Genomed (Warsaw, Poland).

### 2.3. Statistical Analysis

The prevalence of TBPs in ticks was calculated according to the following formula [33]:

$$\mathrm{Prevalence}=\frac{number\;of\;infected\;tick\;specimens}{number\;of\;ticks\;examined}\times 100\%$$

## 3. Results 

**Figure 1:**
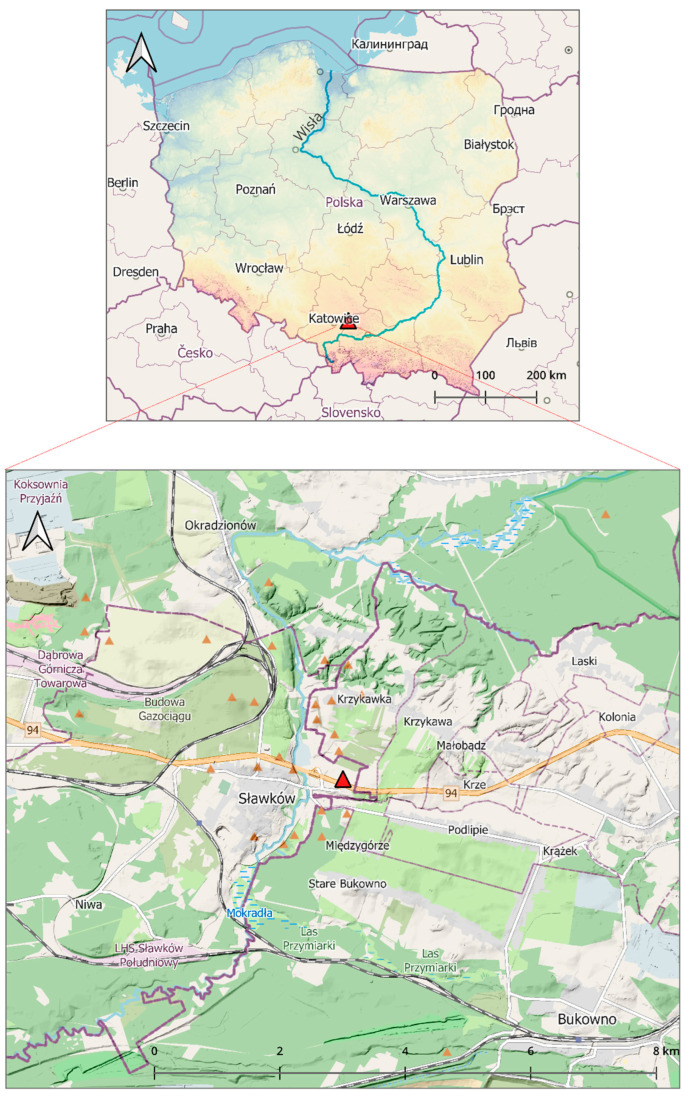
Geographical location of the new endemic locality of *Dermacentor reticulatus* ticks to the west of the Vistula River (Wisła in Polish) in Sławków, Upper Silesia, central–southern Poland (50°30′19.8′′ N; 19°40′72.6′′ E) (prepared by Marcin Wasilewski, marcinwasilewski.eu on the basis of OpenStreetMap; © authors OpenStreetMap).

**Figure 2:**
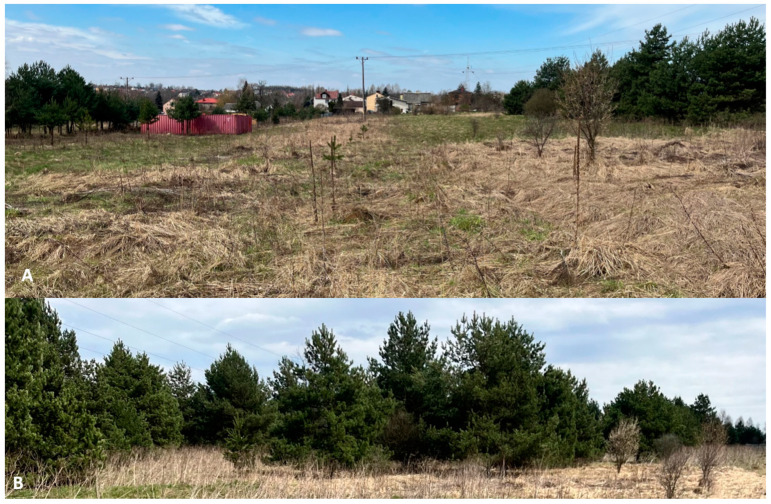
Sites of the occurrence of *Dermacentor reticulatus* in Sławków, Upper Silesia, central–southern Poland (50°30′19.8′′ N; 19°40′72.6′′ E). (**A**) Meadow habitat, (**B**) ecotone habitat.

In this survey, a new locality of *D. reticulatus* occurrence was confirmed in an area regarded as a *Dermacentor*-free zone in central–southern Poland (Figure 1). Ticks of this species were mainly collected from open areas overgrown with grasses near residential buildings (Figure 2). In total, 65 ticks, including 48 *D. reticulatus* and 17 *I. ricinus* adults, were collected from the vegetation in the study area. Only adults of both tick species were collected (Table 2).

On the basis of the scale proposed by Supergan and Karbowiak [27], the risk of *D. reticulatus* tick attack was assessed as high in the open meadow habitat (26–50 adult ticks collected by one person per 1 h) and as moderate in the ecotone habitat (11–25 adult ticks, respectively). In turn, the risk of *I. ricinus* attack was higher in the transition zone between the meadow and the forest, where it was rated as middle (11–25 ticks collected by one person per 1 h) in contrast to the low risk in the area open patch (4–10 ticks, respectively).

In total, *Rickettsia* spp. were detected in 10/48 (23.8%) of the *D. reticulatus* adults. These pathogens were identified in 7/23 (30.4%) males and only 3/25 (12.0%) females. None of the other studied pathogens were present in the analyzed *D. reticulatus* ticks.

In turn, the presence of *B. burgdorferi* s.l., *Babesia* spp., and *Rickettsia* spp. was shown in *I. ricinus*. In total, 13 monoinfections with *B. burgdorferi* s.l., 2 coinfections with *B. burgdorferi* s.l. and *Babesia* spp., and 1 coinfection with *B. burgdorferi* s.l. and *Rickettsia* spp. were detected. This spirochete was found in 13/17 (75.5%) adults of *I. ricinus*. *B. burgdorferi* s.l. was detected in 6/10 (60.0%) *I. ricinus* females and in 7/7 (100.0%) males. In turn, the coinfection with *B. burgdorferi* s.l. and *Babesia* spp. was detected in only 2/10 (20.0%) females of this tick species. The coinfection with *B. burgdorferi* s.l., and *Rickettsia* spp. was found in 1/10 (10.0%) *I. ricinus* females. No *Anaplasma phagocytophilum* or *Bartonella* spp. were detected in the *I. ricinus* ticks.

The analysis of the amplicon sequencing data revealed species diversity among the *Babesia* spp. detected in the *I. ricinus* ticks. *Babesia microti* was identified in one female. The sequence exhibited 100% identity with the sequence of *B. microti* (sequence ID: MK609547.1 human blood, Singapore pathogen imported from the US). In turn, *Babesia venatorum* EU01 was present in another female of the *I. ricinus*. In this case, the sequence exhibited 100% identity with the sequence of *Babesia* sp. “*venatorum*” (sequence ID: MG344777.1 cervid Czech republic, KM289157.1 *Ixodes ricinus* Spain, GQ888709.1 the Netherlands—reindeer, HM113372.1 *Ixodes ricinus* Italy).

## 4. Discussion

The progressive expansion of *D. reticulatus*, a vector with unique adaptive abilities, has been observed throughout Europe in recent decades [6,7,8]. In Poland, where the so-called *Dermacentor*-free zone is found, the increase in the distribution range of this species is regularly investigated, and new endemic locations of this tick species have been described [3,18,23,34,35,36]. Mierzejewska et al. reported 21 new locations of this tick species on the west side of the Vistula River and 22 locations in western Poland [23]. These researchers found *D. reticulatus* in Wielkopolskie, Kujawsko-Pomorskie, and Łódzkie Provinces. In turn, Karbowiak and Kiewra discovered the presence of *D. reticulatus* in the natural habitat of Lower Silesia (south-western Poland) [35]. Further studies confirmed the permanent occurrence of *D. reticulatus* in this area; therefore, this tick species can be regarded as a typical element of the fauna in this region of Poland [17,36]. Similarly, faunistic studies conducted in Lubuskie Province showed new areas of the occurrence of this tick species in western Poland [24,34]. To date, the area of Upper Silesia has been considered *D. reticulatus*-free, as this tick has not been found in samples collected from vegetation in this region. Research on the distribution of this tick species conducted in 2012–2014 in some areas of this province gave negative results [27]. There were also no reports of the permanent presence of *D. reticulatus* in the neighboring provinces in the west (Opolskie Province) and the east (Małopolskie Province). Currently, only three incidents of single infestations of *D. reticulatus* species in dogs have been recorded in Upper Silesia [37,38]. However, in studies of this type, it is difficult to unequivocally confirm the constant presence of ticks in the local environment, and it is impossible to determine the exact place of their origin. In turn, the results presented in this study verify for the first time the occurrence of a *D. reticulatus* population in a new locality in this part of Poland. Unexpectedly, the relatively large numbers of the *D. reticulatus* specimens collected from vegetation indicate that this is a new endemic area of the occurrence of this tick species. In addition, in this newly discovered location, *D. reticulatus* has been reported to outnumber *I. ricinus*, which, except for in eastern Poland, is relatively rare even where tick habitats have been known for a long time [5,6]. Similar results were obtained in research conducted in eastern and central Poland, showing the dominance of *D. reticulatus* over *I. ricinus* in open areas where these two tick species were sympatric [8]. *Dermacentor reticulatus* is characterized by a wide spectrum of temperature and humidity tolerance, which has been confirmed in both laboratory and field studies [12,22,39,40,41]. Nevertheless, the mosaic landscape probably supports increased animal mobility, which in turn results in the creation of routes used by the hosts of these ticks [21]. The appearance of *D. reticulatus* in the analyzed locality is probably associated with the presence of migration routes for tick hosts, mainly Artiodactyla and Canidae, which can transport ticks over long distances [22,42,43,44,45].

The molecular analysis of *D. reticulatus* adults conducted to detect the occurrence of five TBPs in sites located in other regions indicated the presence of a wide TBP spectrum, including TBEV, *Rickettsia* spp., *A. phagocytophilum*, *Bartonella* spp., *B. burgdorferi* s.l., *Borrelia afzelii*, *B. canis*, *B. microti*, *B. venatorum*, *B. vogeli*, *Francisella*-like endosymbionts, and *Toxoplasma gondii* [8,22,24,46,47,48,49]. The PCR analysis of the studied *D. reticulatus* specimens gave positive results only in the case of *Rickettsia* spp. The prevalence of these pathogens was commonly noted in *D. reticulatus* from eastern Poland; for example, Błaszkiewicz identified *Rickettsia* spp. in 38 out of 100 adult ticks collected from vegetation, which made up 14% of females and 62% of males [22]. A comparable prevalence of tick-borne rickettsiae in questing *D. reticulatus* was also reported from Lublin Province by Wójcik-Fatla et al. [50]. The researchers identified the *Rickettsia raoultii* etiological agent of tick-borne lymphadenopathy in 280 of 528 adult ticks (53.8%), which made up 53.8% of males and 52.5% of females collected with the flagging method. In a study on the distribution and epidemiological role of *D. reticulatus* conducted in south-eastern Poland (Subcarpathian region), including extensive screening for the presence of TBPs, a high prevalence of *Rickettsia* spp. in adults of this tick species was demonstrated. In the group of 120 tested individuals, *R. raoultii* was detected in 69 ticks (57.5%), i.e., in 59.6% of *D. reticulatus* females and 55.1% of males, while *R. helvetica* was detected in 0.8% of the ticks, i.e., in one female [51].

In Central Europe, i.e., the Czech Republic, Slovakia, and Hungary, the mean prevalence of *Rickettsia* species in *D. reticulatus* adults assessed by Balážová et al. was 47.9%, with no significant differences between sexes [52]. The presence of rickettsiae in tick females and males increases the risk of infection with this pathogen in hosts present in tick habitats. Animals and, less frequently, humans are parasitized by specimens of both sexes [53].

The presence of both monoinfection with *B. burgdorferi* s.l. and coinfections with *B. burgdorferi* s.l. and *Babesia* spp., as well as *B. burgdorferi* s.l. and *Rickettsia* spp., was detected in the sympatric *I. ricinus* ticks collected in the study area. The percentage of *I. ricinus* infected with *B. burgdorferi* s.l. in southern Poland varies from 0% in some areas of Kraków–Częstochowa Upland to even 62% in some regions of Beskid Żywiecki [54,55]. The prevalence of TBPs confirmed in this study was higher than that shown by Asman et al. [55] and much higher than in other areas of southern Poland, where the number of infected ticks ranged from 4.5% to 15.0% [56,57]. Such a high percentage of *I. ricinus* ticks infected with *B. burgdorferi* s.l. may have resulted from the small number of tested ticks, but it undoubtedly indicates a high potential risk of infection with this pathogen in the studied area.

Reports suggest that concurrent Lyme disease and babesiosis influence the severity of the disease by modifying the course and clinical picture of the polymicrobial infection [58,59]. Studies conducted in various regions of Poland showed that coinfections with *B. burgdorferi* s.l. and *B. microti* were usually detected in a low percentage of examined *I. ricinus* ticks and varied from 0.6 to 2.0% in eastern Poland, 0.3% in northern Poland, to 0.6% in north-western Poland. Moreover, this co-existence was noted more frequently in adult ticks, mainly females, than in nymphs [60,61,62]. The long-term study on the occurrence of both these pathogens and their co-existence in ticks in Poland conducted by Pawełczyk et al. showed that *B. microti* appeared more often in ticks infected with *B. burgdorferi* s.l. [53]. However, in addition to the effect of the species, the diversity of the profile of pathogens infecting ticks may also be related to the biotic and abiotic factors prevailing in their natural habitats [63,64].

The sequencing analysis of *B. microti* showed that this strain represented the US type and was noted in humans who traveled to the US [65]. In turn, the sequence of *Babesia venatorum* EU1 exhibited 100% identity with the sequence of *Babesia venatorum* EU1 isolated from juvenile *Rangifer tarandus* with babesiosis in the Netherlands and *I. ricinus* collected from Italy [66,67]. The present study confirms that *I. ricinus* serves as a vector of these *Babesia* species in the research area.

The knowledge of mutual interactions between *I. ricinus* and *D. reticulatus* co-occurring in the same habitats is still incomplete. It has been demonstrated that the co-feeding of *I. ricinus* and *D. reticulatus* on the same host has a beneficial effect on the reproductive success of both tick species [68]. Pathogen transmission occurring in ticks co-feeding in close proximity on the same host increases the risk of the co-existence of multiple tick-borne pathogens or TBP strains in tick organisms [69]. Research conducted by Buczek et al. also indicates that *I. ricinus* and *D. reticulatus* are engaged in oral–anal contact in the non-parasitic phase of the life cycle, which may possibly support the circulation of TBPs in nature [7]. Since we also observed this type of behavior in ticks sampled in habitats other than those described by Buczek et al., the research on its epidemiological implications seems to be highly justified.

## 5. Conclusions

The presented data confirm the progress in the spread of *D. reticulatus* in southern Poland. The results obtained in the study revealed that, in the studied area in Upper Silesia, there is a new endemic site of *D. reticulatus* occurrence that is sympatric to the *I. ricinus* population and characterized by a high TBP prevalence. Our findings indicate the urgent need to implement effective strategies for the surveillance of TBDs and public campaigns that promote knowledge about the risk of exposure to ticks and tick-borne infections and the preventive measures to avoid tick bites. This seems to be particularly important in areas newly populated by certain tick species, where the risk associated with the occurrence of vector-borne diseases transmitted by those ticks was not previously taken into account.

## Figures and Tables

**Table 1:** Oligonucleotide primes used in detection of *Anaplasma phagocytophilum*, *Bartonella* spp., *Rickettsia* spp., *Babesia* spp., and polymerase chain reaction (PCR) conditions.

Pathogen (Gene Detected)	Primer	Sequence (5′-3′)	Size of Amplification Product [bp]	PCR Conditions [°C/s]			No. of Cycles	Reference
				Denaturation	Annealing	Extension		
*Anaplasma phagocytophilum* (16S rRNA)	ge3a	CACATGCAAGTCGAACGGATTATTC	932	94/30	55/30	72/60	40	[29]
	ge10r	TTCCGTTAAGAAGGATCTAATCTCC						
	ge9f	AACGGATTATTCTTTATAG CTTGCT	546	94/30	55/30	72/60	30	
	ge2	GGCAGTATTAAAAGCAGCTCCAGG						
*Bartonella* spp. (*rpoB*)	1400F	CGCATTGGCTTACTTCGTATG	825	94/30	53/30	72/45	35	[31]
	2300R	GTAGACTGATTAGAACGCTG						
*Rickettsia* spp. (*gltA*)	RpCS.877p	GGGGGCCTGCTCACGGCGG	381	95/20	48/30	60/120	35	[32]
	RpCS.1258n	ATTGCAAAAAGTACAGTGAACA						
*Babesia* spp. (18S rRNA)	Babfor	GACTAGGGATTGGAGGTC	620	94/60	53/45	72/90	35	[30]
	Babrev	GAATAATTCACCGGATCACTC						

**Table 2:** Number of ticks (Acari: Ixodida) collected by flagging sampling in Sławków (central–southern Poland) in mid-April 2023.

Tick Species	Meadow Habitat N (%)			Ecotone Habitat N (%)			Total
	F	M	A	F	M	A	
*Dermacentor reticulatus*	16 (88.9)	14 (93.3)	30 (90.9)	9 (52.9)	9 (60.0)	18 (56.3)	48 (100)
*Ixodes ricinus*	2 (11.1)	1 (6.7)	3 (9.1)	8 (47.1)	6 (40.0)	14 (43.7)	17 (100)
Total *	18 (100)	15 (100)	33 (100)	17 (100)	15 (100)	32 (100)	

N—number of ticks; F—females; M—males, A—adults of both sexes (females and males); * number of adult ticks of each sex in the particular collection site; only adults of both tick species were collected.

## Data Availability

The original contributions presented in the study are included in the article; further inquiries can be directed to the corresponding authors.
